# Supplementary material for: The Analysis of the Editing Defects in the dyw2 Mutant Provides New Clues for the Prediction of RNA Targets of Arabidopsis E+-Class PPR Proteins
Source: Plants (Basel). 2020 Feb 21;9(2):280. doi: 10.3390/plants9020280 (PMC7076377; doi:10.3390/plants9020280)
Supplement: Supplementary file 1 [file plants-09-00280-s001.zip › SupplMaterial/SuppFigures.pdf]

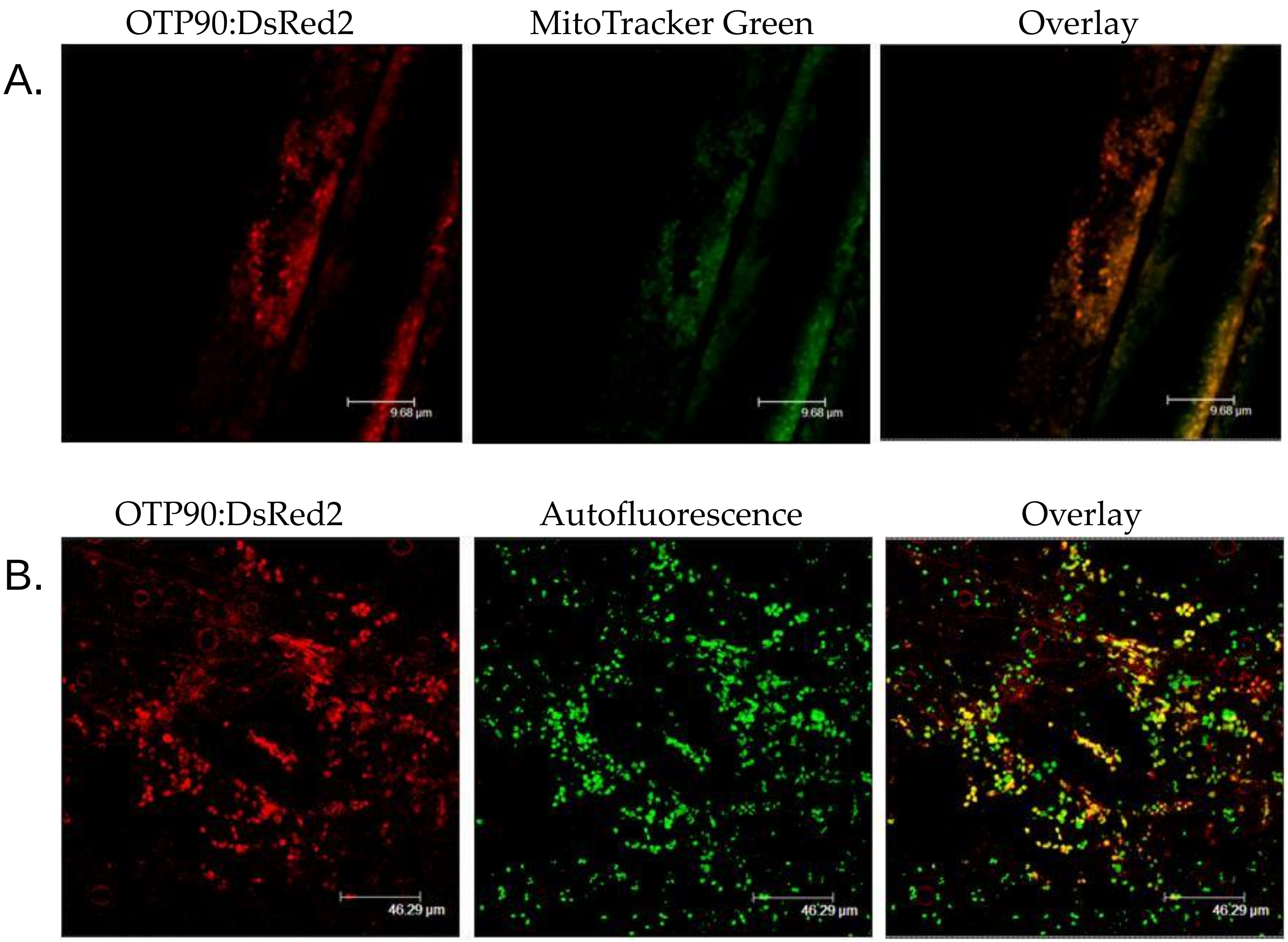

Figure S1. OTP90 is dually targeted to mitochondria and plastids in transgenic *Arabidopsis thaliana* plants.
(a). Roots of Arabidopsis plantlets expressing OTP90:DsRed2 fusion protein stained with MitoTracker Green. The RFP fluorophore (in red) and the MitoTracker Green staining (in green) were simultaneously visualized. Overlay panel shows combined fluorescence.
(b). Cotyledons of Arabidopsis plantlets expressing OTP90:DsRed2 fusion protein. The RFP fluorophore (in red) and the chlorophyll autofluorescence (in green) were simultaneously visualized. Overlay panel shows combined fluorescence.

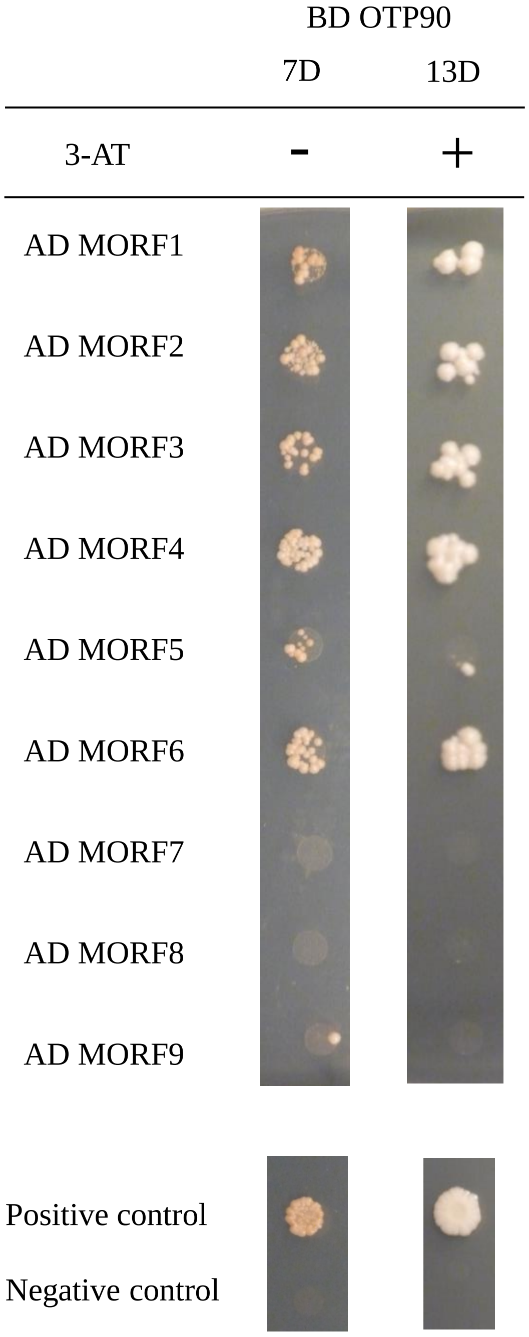

Figure S2. OTP90 interacts with MORF proteins in Yeast two hybrid. The open reading frame for OTP90 was cloned in frame with the binding domain (BD in vector pGBKT7) and was co-transformed into yeast cells with the various MORF proteins in frame with the activation domain (AD) in vector pGADT7. The interactions were evaluated on selective medium with SD-Ade-Leu-Trp-His after 7 days of incubation (7D) and also with2.5 mM of 3-Amino-1,2,4-triazole (3-AT, +) after 13 days of incubation (13D). Positive control is a test of the known protein interaction using the vectors pGBKT7 p35 with pGADT7 T and the negative control using pGBKT7 Lam with pGADT7 T. The results show strong interaction of OTP90 with MORF1, MORF2, MORF3, MORF4 and MORF6.

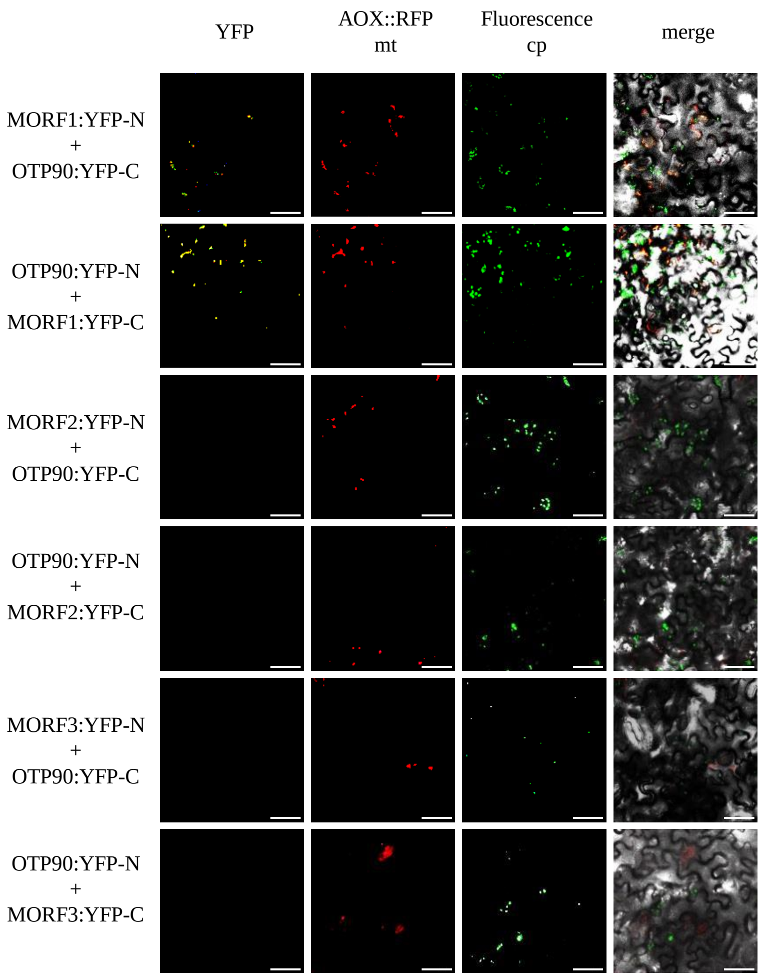

Figure S3. OTP90 interacts with MORF1 in BiFC. BiFC analysis of OTP90 proteins with mitochondrial targeted MORF1, MORF3, and chloroplast targeted MORF2 proteins in tobacco leaves. OTP90 and all MORFs are cloned both in YFP-N and YFP-C vectors. Labels in left sides of each set of panel indicate the various constructions co-transformed into the cells. Labels above each set of panels indicates the channels observed during the experimentation, protein-protein interacting (YFP) signal in yellow, mitochondrial localization control using AOX target sequence fusion with RFP signal in red (mt), chloroplasts control using auto-fluorescent signal in green (cp) and merged of all 3 fluorescent signals with the differential interference contrast image for each construct combination is also showed (merge). The white bars in each panel indicate the scale bar of 50 μm. Except for the combination of OTP90 and MORF1, YFP signals were not detected (results of OTP90 with MORF4, MORF5, MORF6 or MORF9 not shown).

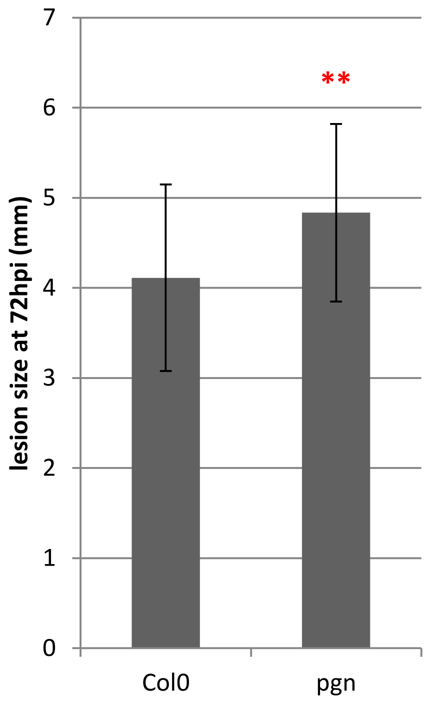

Figure S4. The *pgn* mutant is more sensitive to *Botrytis cinerea*. Lesion size was measured 72 h post inoculation in 2 independent experiments. Asterisks show significant statistical differences between means using Student t-test with α=0.01.

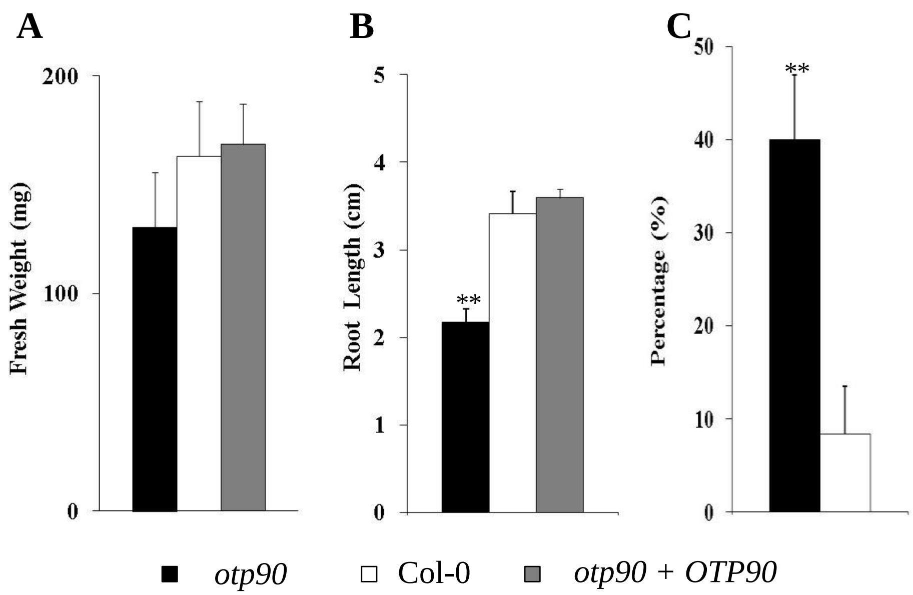

D

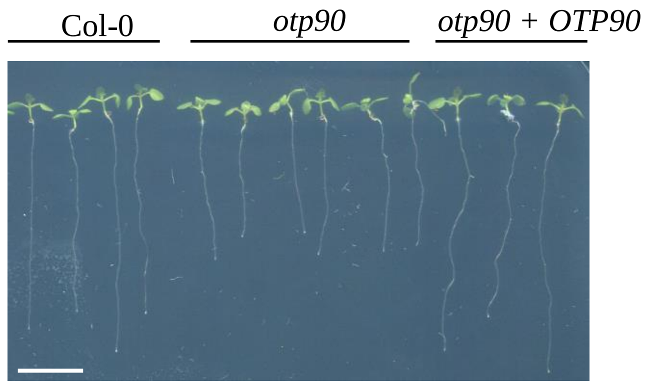

Figure S5. The *otp90* mutant is impaired in root length and seed size.

A. Rosette fresh weights of *otp90* (Black column, n=33), Col-0 (white column, n=33) and *otp90* complemented plants (gray column n=35) are not statistically different 22 days after sowing in soil. Error bars show confidence interval with α=0.05.

B. Seedling root lengths of *otp90* (Black column, n=72) are significantly shorter than that of Col-0 (white column, n=70) and *otp90* complemented line (gray column n=72). Roots were measured 12 days after sowing on Murashine and Skoog medium with 16h/8h light/dark cycles. Error bars show confidence interval with α=0.001 and asterisks show significant statistical differences between means using Student t-test with α=0.001.

C. Percentage of seeds smaller than 25µm among the seeds from *otp90* (Black column, n=4) and Col-0 (white column, n=4) plants. Error bars show confidence interval with α=0.05. Asterisks show significant statistical differences between means using Student t-test with α=0.001.

D. *otp90*, Col-0 and *otp90* complemented line 12 day old- seedlings after germinating on Murashige and Skoog medium with 16h/8h light/dark cycles. White line indicates scale bar of 1cm.

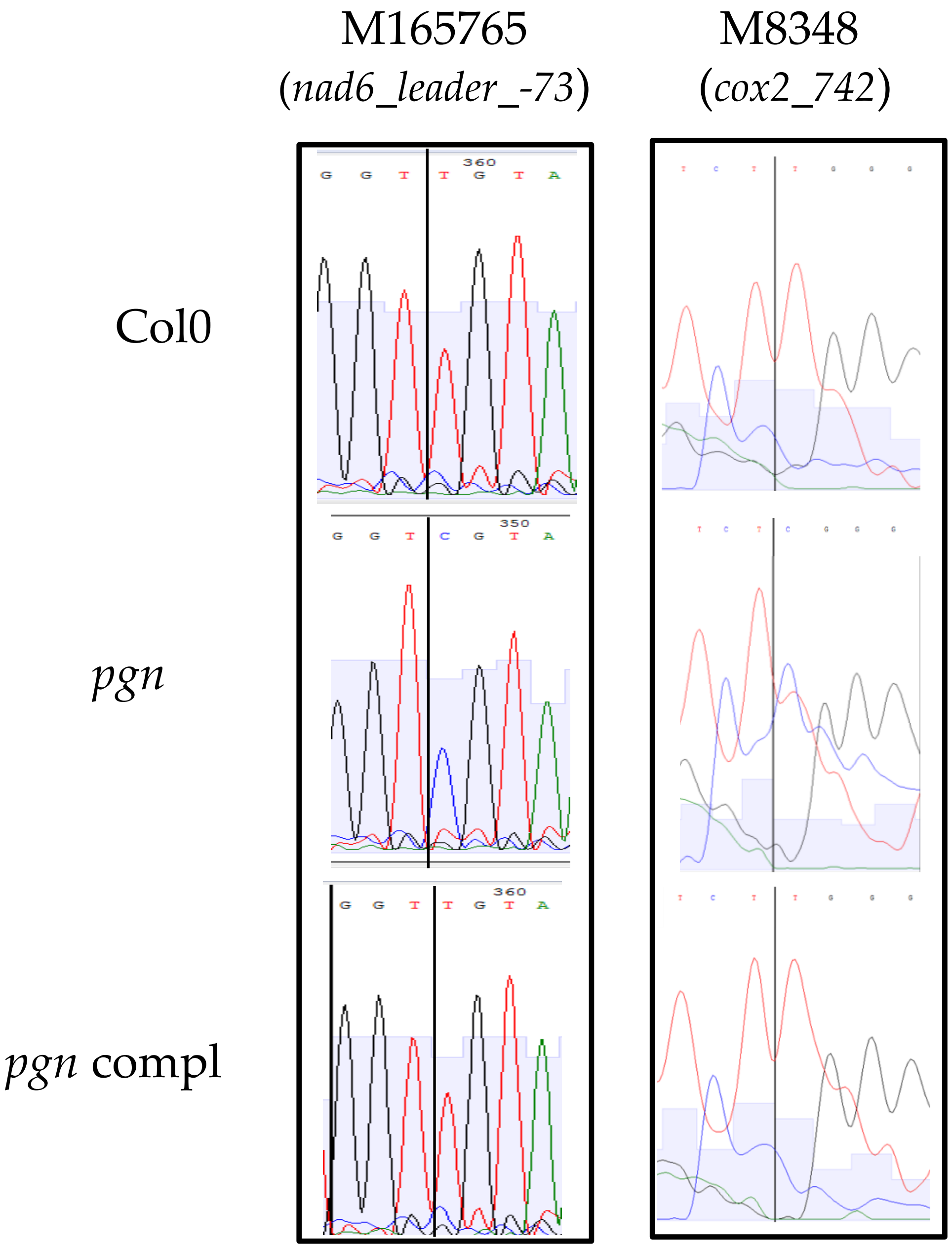

Figure S6. Complementation of the *pgn* mutant for RNA editing defects in 2 mitochondrial sites. Comparison of the cDNA sequence of 2 RNA editing sites from wild type plant Col0, *pgn* and *pgn* complemented by *PGN*.

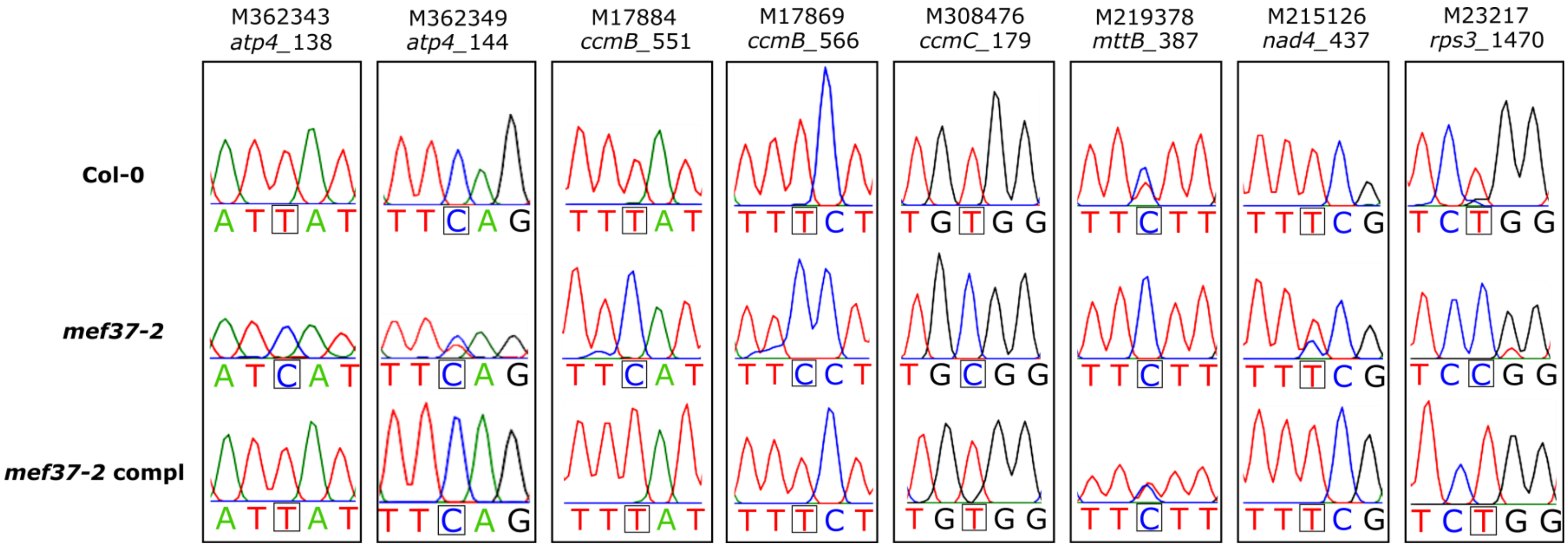

Figure S7. Complementation of the *mef37-2* mutant for RNA editing defects in 8 mitochondrial sites. Comparison of the cDNA sequence of 8 RNA editing sites in the *atp4*, *ccmB*, *ccmC*, *mttB*, *nad4* and *rps3* genes from wild type Col-0, *mef37-2* and *mef37-2* complemented by MEF37.

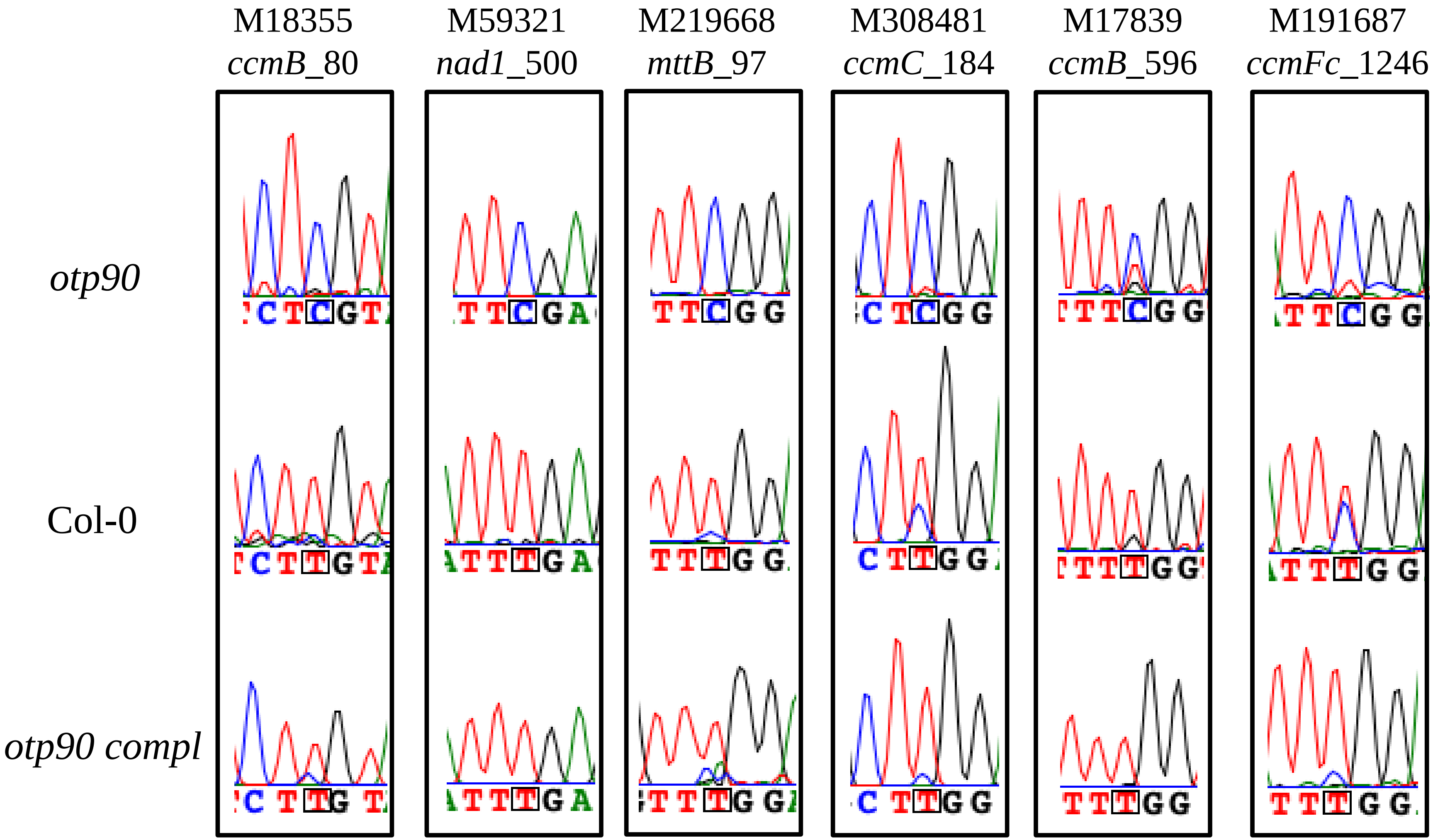

Figure S8. Complementation of the *otp90* mutant for RNA editing defects in 6 mitochondrial sites. Comparison of the cDNA sequence of 6 RNA editing sites in the *ccmFc*, *ccmB*, *nad1*, *ccmC*, *mttB* genes from wild type plant Col0, *otp90* and *otp90* complemented by *OTP90* locus.
